# Characteristics and natural course of hypoechoic thyroid lesions diagnosed as possible thyroid lymphomas by fine needle aspiration cytology

**DOI:** 10.1186/s13044-018-0051-z

**Published:** 2018-05-30

**Authors:** Tomoe Nakao, Mitsushige Nishikawa, Mako Hisakado, Toshihiko Kasahara, Takumi Kudo, Eijun Nishihara, Mitsuru Ito, Shuji Fukata, Hirotoshi Nakamura, Mitsuyoshi Hirokawa, Akira Miyauchi

**Affiliations:** 1 Department of Internal Medicine, Kuma Hospital, Centre for Excellence in Thyroid Cares, 8-2-35 Shimoyamate-dori, Chuo-ku, Kobe, 650-0011 Japan; 2 Department of Diagnostic Pathology and Cytology, Kuma Hospital, Centre for Excellence in Thyroid Cares, 8-2-35 Shimoyamate-dori, Chuo-ku, Kobe, 650-0011 Japan; 3 Department of Surgery, Kuma Hospital, Centre for Excellence in Thyroid Care, 8-2-35 Shimoyamate-dori, Chuo-ku, Kobe, 650-0011 Japan

**Keywords:** Thyroid lymphoma, Hypoechoic thyroid lesion, Regression, Fine needle aspiration cytology, κ/λ deviation

## Abstract

**Background:**

There is little information regarding the natural course of hypoechoic thyroid lesions that are probable or possible thyroid lymphoma based on fine needle aspiration cytology (FNAC) results.

**Methods:**

Sixty-five patients who were diagnosed as probable or possible thyroid lymphoma by ultrasonography (US) and FNAC were investigated. Forty-three patients with strong suspicion underwent thyroid surgery for the diagnosis at our hospital, and 22 patients were followed up with periodic US examination. Thyroid lymphoma was definitely diagnosed in 41 out of 43 patients who underwent thyroid surgery, and such patients were defined as Group A. The outcomes of 22 patients who were followed up without an immediate therapy were analyzed. Their hypoechoic lesions decreased in size (*n* = 10) or disappeared (*n* = 2) in 12 of 22 patients, and such patients were defined as Group B. Patients in Group A and B were compared using the Kuma Hospital-US classification (USC), the diagnostic categories of the Bethesda System for Reporting Thyroid Cytopathology, and the κ/λ deviation of the immunoglobulin light chain in the FNAC specimens. Mann-Whitney *U*-test and chi-squared test (with Yate’s continuity correction) were used to compare the two groups.

**Results:**

The USC of < 3.5 [9/12 (75.0%) in Group B; 10/41 patients (24.4%) in Group A] and the κ/λ deviation ratio of < 3.40 [11/12 (91.7%) in Group B; 17/41 patients (41.5%) in Group A] were significantly more frequent (*p* < 0.01), and the FNAC of ‘benign’ or ‘atypia of undetermined significance or follicular lesion of undetermined significance (AUS)’ with a comment of possible lymphoma [9/12 (75.0%) in Group B; 12/41 patients (29.3%) in Group A] was significantly more frequent (*p* < 0.05) in Group B than Group A.

**Conclusions:**

Our study suggests that some hypoechoic thyroid lesions that are possible thyroid lymphoma based on US and FNAC might decrease in size or disappear during the careful observation.

## Background

Primary thyroid lymphoma is a rare cause of malignancy, accounting for 2–5% of all thyroid malignancies [1] and < 2% of extranodal lymphomas [2, 3]. A rapidly enlarging mass was the most common clinical manifestations in earlier series, but recently small lesions are found at the early stage [4]. It is a potentially lethal disease, but often responds well to appropriate treatments [5]. Thyroid lymphoma is not always diagnosed easily, especially in its early phase, because most of the cases are associated with Hashimoto’s thyroiditis.

Ultrasonography (US) of the thyroid is initially used for the diagnosis of thyroid lymphoma. On US, lymphoma is shown as hypoechoic lesions, but subacute thyroiditis, focal chronic thyroiditis, some thyroid cancers, and metastatic thyroid cancer is also shown as hypoechoic lesions mimicking thyroid lymphoma. Based on US findings of internal echo levels, borders, and posterior echoes, thyroid lymphoma can be classified as the nodular, diffuse, or mixed type [6]. Although the nodular type often resembles follicular tumor or adenomatous nodule and the mixed type often resembles adenomatous goiter on US, these lesions can be often distinguished by an enhancement of posterior echoes. The diffuse type shows homogeneous and hypoechoic internal echoes, but these findings are also typical for severe chronic thyroiditis [6, 7].

Fine needle aspiration cytology (FNAC) is the next diagnostic strategy for thyroid lymphoma, but is challenging, particularly due to its histological similarities with mucosa-associated lymphoid tissue (MALT) lymphoma and chronic thyroiditis [8, 9]. The flow cytometry with CD45 gating on the FNAC specimen can be used to analyze the proportions of lymphocytic cells with κ and λ immunoglobulin light chains. The κ/λ deviation ratio of the light chain assessment is an important criterion for discriminating between polyclonal reactive processes such as chronic thyroiditis and monoclonal lymphomas. Strong deviation in the κ/λ ratio is regarded as suggestive monoclonal growth of lymphocytes, thus indicating thyroid lymphoma [10].

Even with these modalities, a definite diagnosis is not established easily. A surgical interventions is often needed for the histopathological diagnosis, but it might be unnecessary for the benign lesions. This is especially true for the small or moderate-size lesions. Needle biopsy is usually useful for the diagnosis of diffuse large B-cell type lymphoma, but is often insufficient in the case of MALT lymphoma.

We have observed some hypoechoic lesions that were diagnosed as possible thyroid lymphoma based on the FNAC decrease in size or disappear during their clinical courses without definitive treatment. However, there is little information regarding the natural course of such hypoechoic lesions that were possible thyroid lymphoma based on FNAC findings. We conducted the present study to (*1*) clarify the natural course of hypoechoic thyroid lesions that were possible thyroid lymphoma, and (*2*) identify clinical features that might be used to discriminate benign non-progressive lesions from those with progressive character.

## Methods

**Fig. 1:**
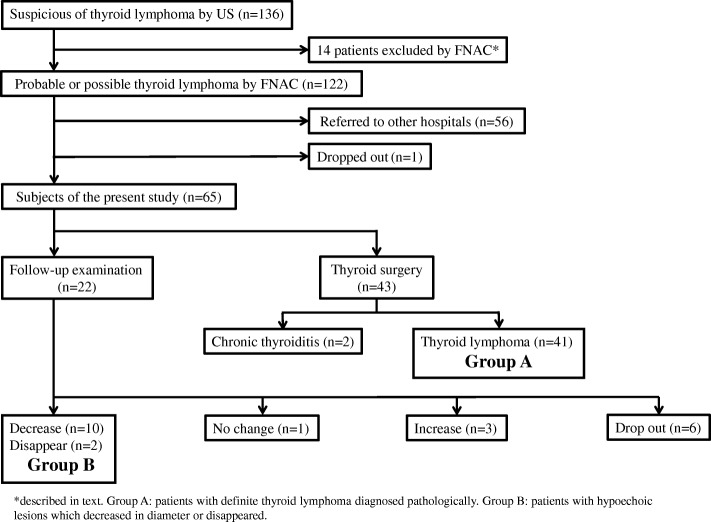
Clinical flow and outcomes of patients with possible thyroid lymphoma based on US and FNAC

Between April 2012 and July 2016, 136 patients were suspected of having thyroid lymphoma on US examination at Kuma Hospital, where approximately 15,000 new patients with thyroid diseases are evaluated annually. US examinations were routinely performed by our well-experienced operators. US was performed using an APLIO 500 TUS-A500 system (Toshiba Medical Systems Co., Ltd., Otawara, Japan) with a PLT-805AT (Toshiba) or PLT-1005BT probe (Toshiba). Patients with systemic lymphoma or previously diagnosed lymphoma were excluded. Of these 136 patients, 122 were diagnosed as having probable or possible thyroid lymphoma by FNAC (Fig. 1). The remaining 14 patients were excluded from the study, because they had other diagnoses based on FNAC findings (eight specimens were normal or benign, one anaplastic carcinoma of the thyroid, one subacute thyroiditis or thyroid papillary carcinoma, and one metastatic renal cell carcinoma; the remaining three specimens were inadequate for diagnosis). Among the 122 patients, 57 were excluded from the study, because 56 were referred to other hospitals for the definitive diagnosis and chemo radiotherapy, and one patient dropped out from the follow-up study. Thus, the remaining 65 patients were investigated in this study. Forty-three patients strongly suspected of having thyroid lymphoma underwent thyroid surgery at our hospital for the definite diagnosis and 22 patients with comments of possible lymphoma on cytology were followed up with periodic US examination for 1–41 (median 11.5) months. Whether surgery or follow-up examination was determined by each doctor in charge based on clinical and radiological findings, including the rapidity of the enlargement of the thyroid mass, and the US and FNAC findings. Of 43 patients who underwent thyroid surgery at our hospital, 41 (95.3%) were diagnosed as definite thyroid lymphoma, and the other two (4.7%) were diagnosed as chronic thyroiditis (Fig. 1). Among the 41 patients who were diagnosed as definite thyroid lymphoma, 34 were diagnosed as MALT lymphoma and seven were diagnosed as diffuse large B-cell lymphoma. Whether the lesions of all 41 patients were limited to the thyroid was not known because all were referred to other hospitals to undergo further diagnostic examinations, including the determination of the disease stage, and treatment.

Hypoechoic lesions of the periodically followed-up 22 patients were analyzed. The US examination was repeated 1–3 month-interval at first and then 6 month-interval when no deterioration was observed. None of 22 patients were treated with steroids or immunosuppressive drugs during the follow-up examination. Other medications or supplementations, or dietary habit including iodine intake were not known in detail. When the maximum diameter of a hypoechoic lesion increased by ≥3 mm, we defined the case as an “increase” of the hypoechoic lesion. When the maximum diameter of a hypoechoic lesion reduced by ≥3 mm, we defined the case as a “decrease.” When the hypoechoic lesions were not detected clearly at follow-up examination, it was defined as “disappeared.” We established this parameter because, in our previous study, plus or minus 2 mm was recognized as an observation variation [11]. In the present study, in some cases in which the entire thyroid volume was observed to have decreased, the maximum diameter of the hypoechoic lesions was measured and analyzed.

**Fig. 2:**
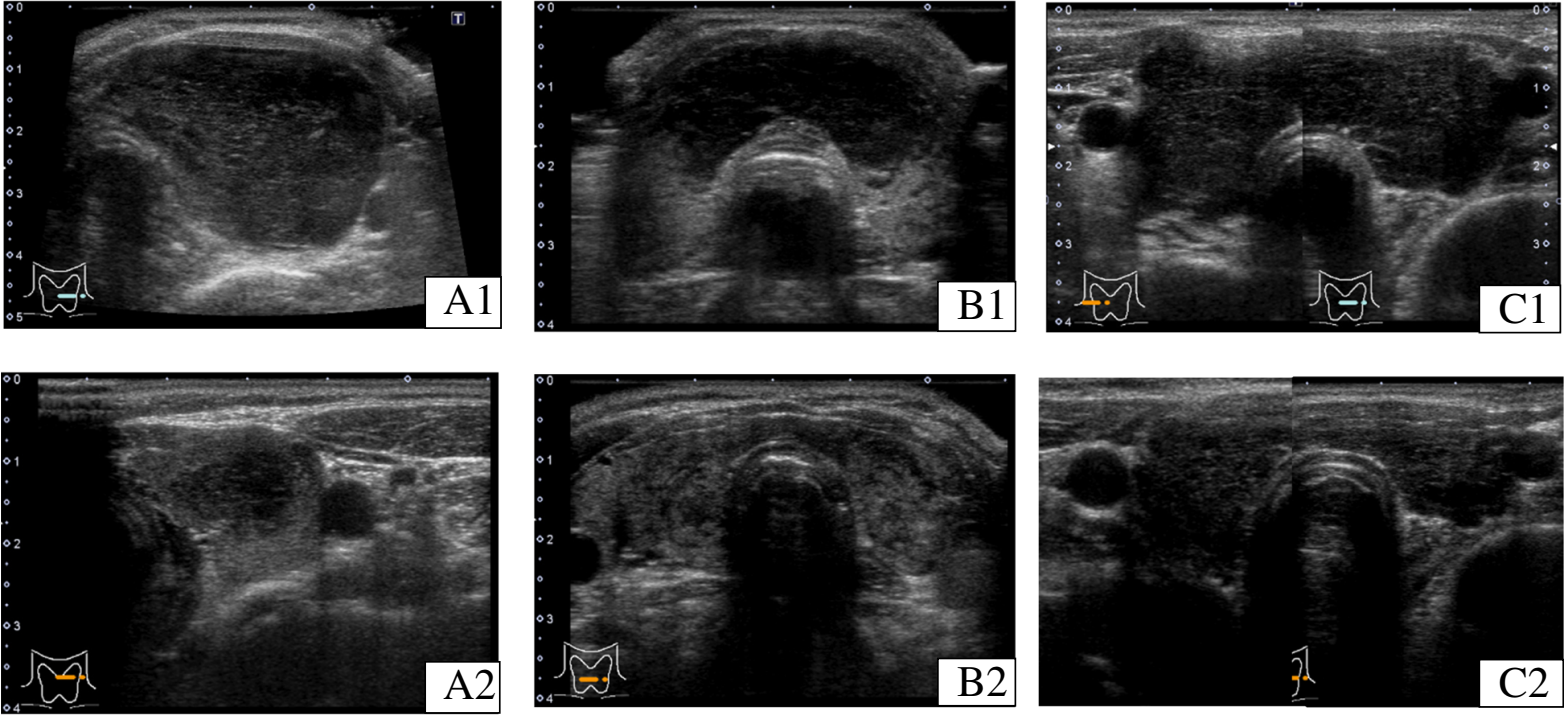
Echograms of three representative patients whose hypoechoic lesions decreased or disappeared on careful follow-up examination. **a** A 74-year-old woman with a nodular hypoechoic lesion at presentation (A1) that had markedly decreased in size one month later (A2) (case No. 9 in Table 2). **b** A 69-year-old woman with a severely hypoechoic lesion involving the both thyroid lobes (B1) that had almost disappeared 10 months later (B2), although the irregularity remained possibly due to underlying chronic thyroiditis (case No. 4 in Table 2). **c** A 53-year-old woman with a diffuse severely hypoechoic lesion involving the whole thyroid (C1) that had markedly decreased in size 27 months later (C2) (case No. 1 in Table 2)

Representative US images showing “decrease” and “disappearance” are shown in Fig. 2. Our hospital uses its own ultrasound classification system for the diagnosis of thyroid nodules, which consists of five US classes (USC) based on the characteristics of thyroid nodules, such as a regular or irregular shape, solid or cystic content, the presence or absence of microcalcifications, extraglandal invasion, and other factors. The classification consists of five classes from 1 to 5. Intermediate classes from class 2 to class 4 (designated as classes 2.5 and 3.5) are also used (Table 1) [12]. Some cases of lymphoma with diffuse or mixed lesion are hardly classified according to USC, because this classification is mainly scored to thyroid nodules. However, in the present study, the US classification was applied to the hypoechoic lesions. The present study was approved by the Institutional Review Board of Kuma Hospital and the Ethics Committee in Kuma Hospital.

**Table 1:** Ultrasonographic classification for thyroid nodule at Kuma Hospital

Class	Description
1	Round or anechoic lesion.
2	Regular-shaped nodule with cystic change. The echo level of solid lesion is similar to that of normal thyroid.
3	Solid and regular-shaped nodule. Internal echo is homogeneous, or may have strong echoes internally or at the capsule.
4	Solid and irregular-shaped nodule. Internal echo is usually low and may have fine strong echogenic spots.
5	Solid and irregular-shaped nodule with extrathyroid extension.

Intermediate classes from class 2 to class 5 (designated as classes 2.5,3.5 and 4.5) are also used

The hypoechoic lesions in 22 patients who were followed up with periodic US examination decreased in size (*n* = 10), disappeared (*n* = 2), showed no change (*n* = 1) or increased in size (*n* = 3). The remaining six patients dropped out from the follow-up examination. Of the three patients whose hypoechoic lesions increased in size, one patient was referred to another hospital and diagnosed as diffuse large B-cell lymphoma; the other two patients were carefully followed up without further progression (Fig. 1).

Forty-one patients with histopathologically diagnosed thyroid lymphoma were defined as Group A, and 12 patients whose hypoechoic lesions decreased in diameter or disappeared as defined Group B (Fig. 1). Their clinical features, the diameter of hypoechoic lesions, US features at the first presentation, and the diagnostic categories of the Bethesda System for Reporting Thyroid Cytopathology (BethSys categories) were compared between Group A and B. Mann-Whitney *U*-test and chi-squared test (with Yate’s continuity correction) were used to compare the two groups.

## Results

Table 2 summarizes the clinical features of 16 patients who underwent follow-up US examinations of hypoechoic lesions that were diagnosed as possible thyroid lymphoma. Thyroid function tests at presentation showed hypothyroid (*n* = 1), subclinical hypothyroid (*n* = 4) and euthyroid (*n* = 11). Some patients were taking levothyroxine (LT4) before and during the follow-up examination. However, whether LT4 supplementation was involved in the decrease or disappearance of the hypoechoic lesions is unclear, because of the small number of the cases. All Group B patients were thyroglobulin antibody (TgAb)- and/or thyroid peroxidase antibody (TPOAb)-positive. As a result of FNAC, ‘atypia of undetermined significance or follicular lesion of undetermined significance (AUS)’ was diagnosed in eight, ‘suspicious for ‘malignancy’ in two, ‘malignancy’ in one, and ‘benign’ in one patient. The pathological report of case 2 in Table 2 said that it was probably benign; however, lymphoma could not be completely excluded. On flow cytometry of the fine needle aspirations, the κ/λ ratio varied from 0.31 to 4.61. When κ/λ ratio was lower than 1.00, we converted κ/λ ratio to λ/κ ratio (deviation ratio) to discriminate between the polyclonal reactive process and the monoclonal reactive process that is characteristic to lymphomas.

**Table 2:** Clinical features of 16 patients who underwent follow-up US examinations of hypoechoic lesions that were diagnosed as possible thyroid lymphoma

Case	Age	Sex	Thyroid function^a^	TgAb (IU/mL)	TPOAb (IU/mL)	Echo pattern of hypoechoic lesions	US class	BethSys^b^	Duration of follow-up (months)	Before (mm)	After (mm)	κ/λ deviation ratio^c^	LT4 supplementation (μg/day)	TSH during follow-up	Outcome
1	53	F	subclinical hypothyroid (with LT4 50 μg/day)	4000≦	535	diffuse	3	AUS^d^	27	61	47	1.21	75	normal	decrease
2	62	F	hypothyroid	4000≦	600≦	diffuse	3.5	benign^e^	30	120	80	1.50	112.5	suppressed	decrease
3	66	F	euthyroid	28	89	nodular	3.5	AUS	12	25	11	4.61	50	normal	decrease
4	69	F	euthyroid	120.7	600≦	nodular	3	AUS	10	37	disappearance	3.14	–	normal	disappearance
5	70	M	euthyroid	54	118	nodular	3	AUS	37	15	12	1.84	–	normal	decrease
6	71	M	subclinical hypothyroid	1171	–	nodular	3	malignancy	6	37	23	3.20	50	normal	decrease
7	72	F	euthyroid	906	–	nodular	2.5	Suspicious for malignancy	3	27	14	2.90	50	normal	decrease
8	74	F	euthyroid	512.9	151	nodular	3	AUS	41	12	disappearance	2.75	–	normal	disappearance
9	74	F	euthyroid	688.8	16	nodular	3	Suspicious for malignancy	1	105	25	1.72	–	normal	decrease
10	74	F	euthyroid	121.8	–	nodular	3	AUS	7	16	9	2.06	75	suppressed	decrease
11	77	F	subclinical hypothyroid	1402	–	diffuse	3	AUS	25	46	40	1.53	50	normal	decrease
12	83	F	euthyroid	89.9	–	nodular	3.5	AUS	6	23	19	3.21	–	normal	decrease
13	66	F	euthyroid	235.5	–	nodular	4	AUS	22	22	22	0.86	75	suppressed	no change
14	46	F	euthyroid	609.6	–	nodular	3	Suspicious for malignancy	7	22	30	–	–	normal	increase
15	87	M	euthyroid	750.4	≦16	nodular	3	Suspicious for malignancy	6	19	23	4.09	–	normal	increase
16	94	F	subclinical hypothyroid	586.3	26.5	diffuse	3	Suspicious for malignancy	11	65	160	1.81	62.5	normal	increase

The normal range of TgAb is ≤39.9 U/mL; TPOAb: ≤27.9 U/mL. –: TPOAb not done or no levothyroxine (LT4) supplementation

Intermediate US classes from class 2 to class 4 (designated as classes 2.5 and 3.5) are also used

^a^Thyroid function at the first examination and diagnosis as possible thyroid lymphoma by FNAC

^b^The Bethesda System for Reporting Thyroid Cytopathology

^c^The κ/λ deviation ratio was calculated by λ/κ ratio when the κ/λ ratio was < 1.00

^d^Atypia of undetermined significance or follicular lesion of undetermined significance

^e^Lymphoma cannot be denied

There were no significant differences in age, sex, thyroid function, TgAb, TPOAb, echo pattern of hypoechoic lesions, USC, BethSys, the diameter of hypoechoic lesions, κ/λ deviation ratio, LT4 supplementation or TSH between the patients whose hypoechoic lesions decreased in size or disappeared and patients whose lesions were unchanged or increased in size.

There were no significant differences in patient age, sex, or the diameter of the hypoechoic lesions between Groups A and B (Mann-Whitney *U*-test) (Table 3). The USC and the κ/λ deviation ratio were significantly lower in Group B than Group A (*p* < 0.001 and *p* < 0.02, respectively). The USC of < 3.5 [9/12 (75.0%) in Group B; 10/41 patients (24.4%) in Group A] was significantly more frequent (*p* < 0.01), and the FNAC finding of ‘benign’ or ‘AUS’ [9/12 (75.0%) in Group B; 12/41 patients (29.3%) in Group A] was significantly more frequent (*p* < 0.05) in Group B than Group A (Tables 4 and 5). Lastly, the κ/λ deviation ratio of the immunoglobulin (Ig) light chain in the FNAC specimens of < 3.40 [11/12 (91.7%) in Group B; 17/41 patients (41.5%) in Group A] was significantly more frequent in Group B than Group A (*p* < 0.01) (Table 6).

**Table 3:** Comparison of clinical features of Group A and B

	Group A (*n* = 41)	Group B (*n* = 12)	*p*-value
Age (year)	67 (48–91)	72 (53–83)	0.35
Sex (Male, number)	11	2	0.48
Diameter of hypoechoic lesions (mm)	35 (16–75)	38 (12–120)	0.51

Group A is the patients with thyroid lymphoma diagnosed histopathologically following thyroid surgery. Group B is the patients whose hypoechoic lesions decreased or disappeared. Data are median and (ranges). The *p*-values were calculated by Mann-Whitney *U*-test

**Table 4:** Comparisons of US classification between the Group A (*n* = 41) and Group B (*n* = 12)

US class	Group A	Group B
2.5	1	1
3	9	8
3.5	9	3
4	21	0

*P* < 0.01

Classes 2.5 and 3.5 designate intermediate classes between 2 &3 and 3 & 4, respectively

**Table 5:** Comparisons of the diagnostic categories of the Bethesda System for Reporting Thyroid Cytopathology between the Group A (n = 41) and Group B (n = 12)

Diagnostic categories	Group A	Group B
Benign	1	1
AUS	11	8
Suspicious for malignancy	22	2
Malignant	7	1

*P* < 0.02

*US*: ultrasound. *AUS*: atypia of undetermined significance or follicular lesion of undetermined significance

Benign: benign, but with the comment that lymphoma cannot be denied

**Table 6:** Comparisons of Groups A and B regarding the κ/λ deviation ratio of Ig light chain

κ/λ deviation ratio	Group A	Group B	Total
≥3.40	24	1	25
< 3.40	17	11	28
Total	41	12	53

The κ/λ deviation ratio of < 3.40 was significantly more frequent in Group B compared to Group A (*p* < 0.01, chi-square test (with Yate’s continuity correction)). When the κ/λ deviation ratio was lower than 1.00, we converted it to the λ/κ ratio (deviation ratio)

## Discussion

The characteristics and natural course of hypoechoic lesions that were diagnosed as possible thyroid lymphoma based on the FNAC findings were investigated. Careful US examinations were repeatedly performed in 22 of 65 patients, and hypoechoic lesions decreased in diameter in 10 patients (10 of the 22; 45.5% or 10 of 65 patients; 15.4%), and disappeared in two patients (two of 22; 9.1% or two of 65 patients; 3.1%).

The USC and the κ/λ deviation ratio were significantly lower in Group B than Group A, and the FNAC finding of ‘benign’ or ‘AUS’ was significantly more frequent in Group B than Group A. Although thyroid lymphoma typically presents with a rapidly enlarging neck mass leading to compressive symptoms [4], some hypoechoic lesions diagnosed as possible thyroid lymphoma might regress in their natural courses in the present study.

Several studies have demonstrated that *Helicobacter pylori* infection is associated with low-grade gastric MALT lymphoma, and that the eradication of *Helicobacter pylori* can cause histological regression of the lymphoma [13, 14]. Uohashi et al. reported that a hypoechoic lesion in the right lobe disappeared spontaneously after being diagnosed pathologically as non-Hodgkin’s lymphoma following a contralateral lobe resection, and suggested spontaneous regression of acute inflammation [15]. Okamoto et al. reported a patient whose primary thyroid lymphoma of cytotoxic T-cell origin regressed spontaneously [16]. Although its cause remains unknown, it may be attributable to an association of acute inflammation [17, 18], and immune mechanisms such as those involving T-helper cells or natural killer cells of the peripheral blood [19].

However, to our best knowledge, there has been no report that the natural course of hypoechoic lesions suspected of being possible thyroid lymphoma was studied extensively. With the combination of US, FNAC, and CD gating analysis for κ/λ deviation, candidate for the surgery could be accurately selected in 41 out of 43 patients (95%) as demonstrated in Group A. However, the management of not strongly suggestive of lymphoma may be controversial. In the present study, the hypoechoic lesion decreased in size in 10, and disappeared in two out of 22 patients, suggesting the careful observation for such lesions.

The limitation of the present study concerns the retrospective analysis that may eliminate the ability to obtain the strong conclusions. Selection of open surgery or careful observation depends on various factors including the results of FNAC and patients’ previous courses. Therefore, the proper diagnostic rate of Group A was higher than in that of Group B, and the selection bias might cause the difference of the course of the two groups. The nature of the hypoechoic lesions that regressed during the follow-up examination is unclear in the present study, because open biopsies were not performed in these patients. Some could be actually low-grade thyroid lymphoma, but others could be focal or regional lymphocytic thyroiditis, atypical subacute thyroiditis, or other etiology-unknown lesion(s). Further prospective studies including histopathology and factor(s) involved in the regression or progression of the lesions are needed to investigate the true nature of such lesions, and to clarify the best method of managing such patients.

Hypoechoic lesions suspected of having possible lymphoma decreased in size or disappeared in 12 out of 22 patients (55%), and such a regression should be emphasized in the present study. Because most of these lesions cannot be diagnosed definitely by core needle biopsy, open surgery might be considered. But unnecessary surgeries for benign lesions might be avoided.

## Conclusion

Our study suggests that some hypoechoic thyroid lesions that are possible thyroid lymphoma based on US and FNAC might decrease in size or disappear during the careful observation. A careful observation before surgery is suggested for those lesions such as USC < 3.5, a κ/λ deviation ratio < 3.4, and the FNAC classification of ‘benign’ or ‘atypia of determined significance or follicular lesions of undetermined significance’. However, an open biopsy or thyroid surgery should be considered in case of the increasing lesion in diameter on follow-up US examination.

## Abbreviations

AUS: Atypia of undetermined significance or follicular lesion of undetermined significance
BethSys categories: Bethesda System for Reporting Thyroid Cytopathology
FNAC: Fine needle aspiration cytology
Ig: Immunoglobulin
MALT: Mucosa-associated lymphoid tissue
TgAb: Thyroglobulin antibody
TPOAb: Thyroid peroxidase antibody
US: Ultrasonography
USC: US classification
